# Mediterranean Diet and Other Dietary Patterns in Primary Prevention of Heart Failure and Changes in Cardiac Function Markers: A Systematic Review

**DOI:** 10.3390/nu10010058

**Published:** 2018-01-10

**Authors:** Karina Sanches Machado d’Almeida, Stefanny Ronchi Spillere, Priccila Zuchinali, Gabriela Corrêa Souza

**Affiliations:** 1 Heart Failure and Transplant Group–Hospital de Clínicas de Porto Alegre, Porto Alegre 90035-003, Brazil; karinasmdalmeida@gmail.com (K.S.M.d.); spillerestefanny@gmail.com (S.R.S.); priccizuchi@gmail.com (P.Z.); 2 Nutrition Graduate Course, Universidade Federal do Pampa, UNIPAMPA, Itaqui 97650-000, Brazil; 3 Multiprofessional Health Residency Program/Hospital de Clínicas de Porto Alegre—HCPA, Porto Alegre 90035-003, Brazil; 4 Department of Nutrition, School of Medicine, Universidade Federal do Rio Grande do Sul, Porto Alegre 90035-003, Brazil; 5 Post-Graduation Program in Food, Nutrition and Health, School of Medicine, Universidade Federal do Rio Grande do Sul, Porto Alegre 90035-003, Brazil

**Keywords:** heart failure, dietary patterns, mediterranean diet, DASH

## Abstract

Background: Heart failure (HF) is a complex syndrome and is recognized as the ultimate pathway of cardiovascular disease (CVD). Studies using nutritional strategies based on dietary patterns have proved to be effective for the prevention and treatment of CVD. Although there are studies that support the protective effect of these diets, their effects on the prevention of HF are not clear yet. Methods: We searched the Medline, Embase, and Cochrane databases for studies that examined dietary patterns, such as dietary approaches to stop hypertension (DASH diet), paleolithic, vegetarian, low-carb and low-fat diets and prevention of HF. No limitations were used during the search in the databases. Results: A total of 1119 studies were identified, 14 met the inclusion criteria. Studies regarding the Mediterranean, DASH, vegetarian, and Paleolithic diets were found. The Mediterranean and DASH diets showed a protective effect on the incidence of HF and/or worsening of cardiac function parameters, with a significant difference in relation to patients who did not adhere to these dietary patterns. Conclusions: It is observed that the adoption of Mediterranean or DASH-type dietary patterns may contribute to the prevention of HF, but these results need to be analyzed with caution due to the low quality of evidence.

## 1. Introduction

Heart Failure (HF) is considered a complex clinical condition that compromises the heart’s ability to deliver oxygen properly to tissues. Generally, HF results from structural and/or functional dysfunction of the heart, which compromises its ability to fill itself with blood or to eject blood [1].

Although improvements in the prevention and treatment of HF in the last decades [2] have been observed, mortality rates in this population remain high, with 32.9% of patients with decompensated HF dying within one year of their discharge from hospital [3]. In a study including 6597 patients with acute heart failure attended at 34 Spanish Emergency Departments from 2007 to 2014, the mortality rate was 2.8% within 3 days after hospitalization [4].

According to estimates by the World Health Organization, about three-quarters of deaths from cardiovascular disease (CVD) can be prevented by measures of prevention and control of risk factors that involve changes in lifestyle [5]. The most important behavioral risk factors of heart disease and stroke are unhealthy diet, physical inactivity, tobacco use, and harmful use of alcohol [6].

Since the 1990s, with the transition from diets based on specific nutrients to an approach based on dietary patterns in CVD, promising new data emerged from randomized trials. A dietary approach to nutritional interventions in CVD had proved to be an effective strategy resulting in strong and tangible results, indicating that synergistic effects of food combined into a dietary pattern provide the maximum benefit obtainable from nutrition [7].

The optimal dietary pattern to reduce CVD is one that emphasizes whole grains, fruits and vegetables, legumes, nuts, fish, poultry, and moderate dairy and heart-healthy vegetable oil intake; this pattern will likely reduce the CVD risk by about one-third. This healthy dietary pattern needs also to be low in refined grains, added sugars, trans fats, sugar sweetened beverages, and red and processed meats [8]. Dietary patterns such as the Mediterranean and Dietary Approaches to Stop Hypertension (DASH) can be palatable and relatively easy to adhere to, and are consistent with dietary recommendations for cardiovascular health [9,10]. The traditional Mediterranean-type diet, including plant foods and an emphasis on plant protein sources, provides a well-tested healthy dietary pattern to reduce CVD [8]. The DASH diet has also attracted much attention due to its beneficial effects on blood pressure. This pattern emphasizes consumption of fruits, vegetables, whole grains, poultry, fish, nuts, and low-fat dairy products and minimizes consumption of red meat, sweets, and sugar sweetened beverages [11,12,13]. It is well known that high blood pressure is associated with functional changes in the heart and blood vessels, including impaired left ventricle (LV) function [14,15,16]. Consequently, this suggests that a DASH-type diet could also be of benefit in reducing the risk of LV dysfunction.

Moreover, adherence to these healthy patterns has been shown to be associated with lower CVD risk and markers [17] such as hypertension, hypercholesterolemia, obesity, and diabetes in previous analyses as well as consumption of fatty acids [18].

Although there is evidence that supports the protective effect of certain healthy dietary patterns in CVD, to our knowledge, there is no systematic review or meta-analysis that investigates the protective effect of dietary patterns specifically on primary prevention of HF. Thus, our interest is justified to investigate systematically this effect and to assess the magnitude of the relationship between six dietary patterns and primary prevention of HF.

## 2. Materials and Methods

This systematic review was conducted according to The Cochrane Collaboration [19] and the Preferred Reporting Items for Systematic Reviews and Meta-Analyzes (PRISMA-P Statement) [20]. This study is registered in the International Prospective Register of Ongoing Systematic Reviews and Meta-Analyzes (PROSPERO) under number 42016050355.

### 2.1. Search Strategy and Study Selection

We found studies by searching the following data sources: Medline (accessed by Pubmed), Embase, and Cochrane Central Register of Controlled Trials database up to 20 May 2017. No filter or limitation was used during the search. References lists from selected studies were manually scanned to identify any other relevant studies.

In order to obtain a high-sensitivity strategy, the following search terms were used, both as free-text and subject headings: “dietary pattern”, “diet Mediterranean”, “dash diet”, “diet vegetarian”, “diet Paleolithic”, “diet fat-restricted”, “diet, carbohydrate-restricted” and “heart-failure”.

### 2.2. Eligibility Criteria

To assess comprehensively the magnitude of adherence to dietary patterns and primary prevention of HF, studies with healthy or unhealthy adult subjects were included. The studies included data comparing groups regarding adherence to dietary patterns (Mediterranean diet, DASH, low-carb diet, low-fat diet, paleolithic diet, and vegetarian diet). There was no restriction of study designs included. Experimental studies, those that included children, those that did not present dietary patterns, and those in which patients initially had HF were excluded.

### 2.3. Data Extraction

Titles and abstracts were evaluated independently by two reviewers (S.R.S and K.S.A). The Kappa index was calculated to assess the agreement between the two reviewers and any discrepancy was resolved by consensus or by a third reviewer (G.C.S.). The reviewers were not blind to author, institutions, or manuscript journals. Articles that did not provide sufficient information from the title and abstract were included for further evaluation, and reading was done in full. Data extraction and analysis were performed by the same two reviewers. For each study, information on publication data, population characteristics, intervention and comparison group, results, and limitations were extracted. The authors of the abstract were contacted by e-mail to provide more information about their research.

### 2.4. Assessment of Bias Risk and Study Quality

Methodological quality was explored using an approach similar to that recommended by Cochrane Collaboration in risk assessment [19]. Quality assessment and risk bias included specific questions for randomized clinical trials (RCTs) and observational studies. The following dimensions were considered: study design, sequence generation, concealment of allocation, blinding, losses and exclusions, and description of confounding factors. Risk judgment was assessed using pre-specified study criteria and expressed as “low risk of bias”, “high risk of bias”, or “unclear risk of bias”.

## 3. Results

### 3.1. Descriptions of Studies

A total of 1119 studies were found, and after removal of the duplicates, 1050 were screened by title and abstract. After reading the titles and abstracts, only 14 studies were included in the systematic review (Table 1). The agreement between the reviewers was substantial: κ = 0.625 (*p* = 0.02). 

**Figure 1:**
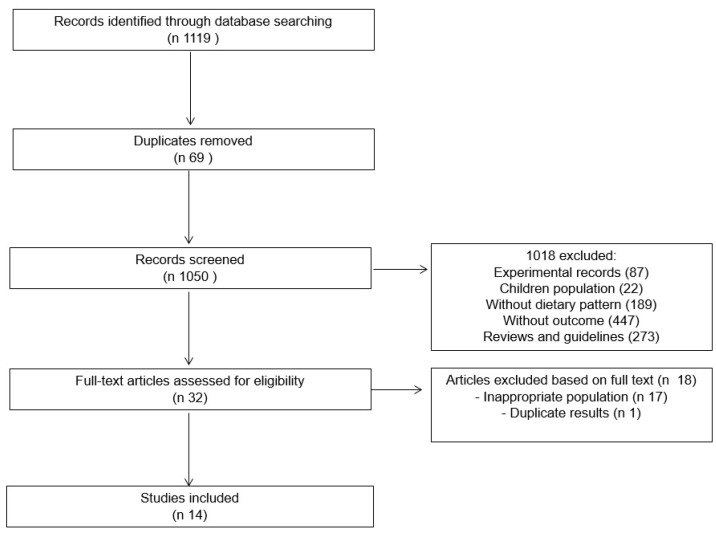
Flowchart for selection of articles.

The study by Larsson et al. [21] was excluded because it was performed with the same cohorts already analyzed in other articles separately. At the end, five RCTs, seven cohort studies, and two cross-sectional studies were used for the systematic review (Figure 1).

A total of 188.470 participants were included in this review. Four RCTs [22,23,24,25] and four cohort studies [26,27,28,29] investigated the effects of adherence to the Mediterranean diet and these were conducted in the United States [22], Spain [23,24], France [25], Germany [26], Greece [27], and Sweden [28,29]. Regarding the DASH diet, three cohort studies [30,31,32] and one cross-sectional study [33] were included, two of which were from the United States [30,33] and two from Sweden [31,32]. Finally, a Swedish RCT investigated the Paleolithic diet [34], and a US cross-sectional study investigated the relationship of vegetarian diet and heart failure [35].

Most studies included patients without previous cardiovascular disease and only two studies were performed in patients after the first infarction. There were no studies that evaluated the adherence of the low-carb and low-fat diets on the incidence of HF or worsening of the parameters of cardiac function. The follow-up period ranged from one to 21 years. Table 1 shows more details about the studies included in the systematic review.

### 3.2. Quality and Publication Bias Assessment

Two RCTs presented a clear description of sequence generation and concealment of allocation. All studies described the losses and exclusions as well as the blindness of the outcome assessor. Regarding blinding of the researchers and participants it was considered as an uncertain risk because it is not possible to blind them. All observational studies performed the analysis describing the confounding factors. Two studies were pointed out with high risk of bias in the item on baseline differences of the participants; both were abstracts (Table 2).

### 3.3. Mediterranean Diet

#### 3.3.1. Definition in the Included Studies

The RCTs included in the review differed in relation to the Mediterranean diet applied to the participants.

In the Papadaki et al. [23] and Fitó et al. [24] studies, the general guidelines of traditional Mediterranean diet (TMD) recommended the use of olive oil in the TMD + olive oil group (≥4 tablespoons/day) for cooking and to taste, vegetables (≥3 servings/week), fish and seafood (≥3 servings/week), and walnuts (≥3 servings/week); and in the TMD + nuts group, a daily portion of 30 g (15 g of nuts, 7.5 g of almonds, and 7.5 g of hazelnuts); white meat instead of red or processed meat, with the last item restricted to <1 portion/day; consumption of “sofrito” (≥2 servings/week), a sauce of tomato, garlic, onion, and aromatic herbs with olive oil to serve with vegetables, pasta, rice and other dishes; and continuous use of red wine for participants who already used it. Consumption of soft drinks (<1 drink/day), bakery products (<3 servings/week) and added fats (<1 servings/day) were discouraged. Participants of the TMD groups received olive oil (15 L for 3 months) or nuts hazelnuts and almonds (1350 g of nuts, 675 g of hazelnuts, and 675 g of almonds for 3 months). To assess adherence, a validated questionnaire of 14 items [36] was used.

In the RCT conducted by Lorgeril et al. [25], no scoring was used and the basic principles of the experimental diet were described in studies previously conducted by his research group. The dietary instructions were personalized and detailed for each patient in the intervention group. These instructions took into account the wishes and gastronomic preferences of the patients, their geographic origin, the patient’s occupation, the number of people in the household, the financial resources of the family, etc. Instructions had to be detailed, easy to comply with, safe, inexpensive, and compatible with the way of living (namely single people, couple, large family, retired, unemployed, still working, etc.), ethnic origin, gender, and religion of each patient. The control group received general guidelines for a prudent diet. Patients were advised to change their regular diet in two steps. In the first step, the cardiologist presented the Mediterranean civilization and explained what Mediterranean diet means. The second step of the intervention was conducted by the study Nutritionist and consisted in showing patients and their families how to adapt their diet to the Mediterranean pattern. The diet consisted of consuming more whole grain breads, root and green vegetables, and fish and consuming less meat and daily fruit consumption as well as replacing pork for poultry and replacing butter for canola oil-based margarine provided by the study since patients did not accept olive oil. Moderate wine consumption was allowed with meals. Adherence to the diet was performed by a food frequency questionnaire and a 24-h recall [37,38].

In the study proposed by Tuttle et al. [22], the main goal of dietary intervention was to reduce calories from saturated fat to <7% and cholesterol intake to <200 mg/day, both in the Mediterranean and in the low-fat diet. In the Mediterranean-style diet, there were additional goals of increasing the intake of omega-3 fatty acids (≥0.75% of calories) and monounsaturated fat (20% to 25% of calories). Both diets recommended an increase in the intake of fresh fruits and vegetables (≥5 servings/day) and whole grains. The Mediterranean-style diet was distinguished by an emphasis on increased consumption of cold water fish (3 to 5 times/week) and olive, canola, and soybean oils.

In the study by Wirth et al. [26] and in both cohort studies performed by Tektonidis et al. [28,29] the modified Mediterranean diet (m-MED) score was adapted from the Mediterranean diet scale proposed by Trichopoulou et al. [39]. The m-MED score included eight items: (1) vegetables and fruits (excluding fruit juices and potatoes); (2) legumes and nuts; (3) unrefined grains (whole wheat bread, crusty bread, oatmeal, and wheat bran) (4) fermented dairy products (cultured milk, yogurt, and cheese); (5) fish; (6) red and processed meat; (7) use of olive oil and/or canola oil; and (8) alcohol. Participants with above-median intake received 1 point for presumed beneficial components: vegetables and fruits, legumes and nuts, unrefined grains, fermented dairy products, and fish; for intake below the median intake they received 0 points. For red and processed meats, the reverse was applied. For alcohol consumption, participants received 1 point if alcohol intake was between 5 and 15 g/day and 0 point if alcohol intake was <5 or >15 g/day. For the quality of the fat, 1 point was assigned to the men who used olive oil and/or canola oil as the main source of fat for cooking or as seasoning, otherwise 0 points. The total m-MED score varied from 0 (low adherence) to 8 (high adherence to the Mediterranean diet).

Chrysohoou et al. [27] describes that adherence to the Mediterranean diet was evaluated by the Med-Diet Score, an instrument already validated, which adds up to 55 points. The instrument evaluates the intake of ten food groups (non-refined cereals and products, fruit, nuts, vegetables, potatoes, dairy and its products, fish and seafood, poultry, red meat or meat products, and use of olive oil) as well as the consumption of alcohol. The diet score was calculated as follows: for consumption of items presumed to be close to the Mediterranean pattern (non-refined cereals and products, fruit and nuts, vegetables, olive oil, nonfat or low-fat dairy, fish, potatoes, and pulses), a score of 0 was assigned when a patient reported no consumption, 1 when reported consumption was 1–4 times/month, 2 for 5–8 times/month, 3 for 9–12 times/month, 4 for 13–18 times/month, and 5 for 18 times/month. In contrast, for consumption of foods presumed not to be consumed on a daily or weekly basis (such as meat or meat products, eggs, poultry, and dairy), reverse scores were assigned (e.g., 0 when a patient reported almost daily consumption to 5 for rare or no consumption). Regarding alcohol intake, a score of 5 was assigned for consumption of 3 glasses/day; 0 for none or consumption >7 glasses/day; and scores of 4, 3, 2, and 1 for the consumption of 3, 45, 6, and 7 glasses/day, respectively. This non-monotonic scoring followed the rationale of the Mediterranean dietary pattern that suggests an intake of 15–30 g ethanol/day. Higher values of this diet score indicate greater adherence to the Mediterranean diet (theoretical range: 0–55).

#### 3.3.2. Mediterranean Diet and HF

In two randomized clinical trials, which assessed Mediterranean diet and incidence of HF, the population consisted of individuals who had already presented with their first ischemic event. Tuttle et al. [22] did not observe a difference in cardiovascular events, including HF, among the groups that received a Mediterranean or low-fat diet, but the patients who received the dietary interventions had a better survival, free of cardiovascular events (85 of 101 patients) than the usual care controls (61 out of 101 patients), with adjusted odds ratios (ORs) of 0.28 (95% CI 0.13–0.63, *p* < 0.002). However, when assessing only the outcome of HF development, there was no significant difference (*p* < 0.25). Lorgeril et al. [25] worked with composite outcomes, which combined cardiac death and nonfatal myocardial infarction (composite outcome 1-CO 1) plus secondary outcomes (unstable angina, stroke, heart failure, pulmonary or peripheral embolism) (composite outcome 2-CO 2). In the Mediterranean-diet group, CO 2 was reduced (27 events versus 90 in the control group, *p* = 0.0001). Regarding HF outcomes, six patients in the intervention group and 11 in the control group developed HF; however, statistics of this comparison are not shown.

A secondary arm pre-specified in PREDIMED (Prevención con Dieta Mediterránea), presented by Papadaki et al. [23], evaluated the incidence of HF in participants with a high risk of cardiovascular disease. Participants were assigned to a low-fat diet or one of two traditional Mediterranean diets (TMD + virgin olive oil or TMD + walnuts). After 4.8 years of follow-up, among the 7403 participants, 29 new cases of HF were observed in the TMD + virgin olive oil group, 33 in the TMD + nut group, and 32 in the control group. There was no significant association with the incidence of HF for TMD + virgin olive oil and TMD + nuts compared to the control group, with a hazard ration (HR) of 0.68 (95% CI 0.41 to 1.13) and HR of 0.92 (95% CI 0.56 to 1.49), respectively.

During analysis of the studies, one RCT that evaluated adherence to the Mediterranean diet with HF-related markers in individuals at high cardiovascular risk was found [24]. Participants were assigned to a low-fat diet (control, *n* = 310) or one of two traditional Mediterranean diets (TMD + virgin olive oil or TMD + nuts). After one year of intervention, both TMD decreased pro-brain natriuretic peptide (BNP), with changes reaching significance versus control group (*p* < 0.05). Oxidized low density lipoprotein (LDL) decreased in both TMD groups, and the decrease in the TMD + olive oil group was significantly higher than that in the control group (*p* = 0.004). Changes in lipoprotein (a) after TMD + olive oil were significantly lower than that in the control group (*p* = 0.038).

Regarding the cohorts that evaluated the adherence of the Mediterranean diet, three consisted of healthy individuals [26,28,29] and one included individuals with coronary artery disease [27].

In the German cohort composed of 24,008 healthy middle-aged participants, it identified 209 cases of HF in 8.2 years. After multivariate adjustment, the association between adherence to the Mediterranean diet and HF was not significant, with a HR of 0.82 (95% CI 0.64 to 1.05) [26].

In the postmenopausal women’s cohort, 1648 cases of HF were recorded, 244 of which were in the quartile with the highest adherence to the diet. The high adherence to the Mediterranean diet, compared to the groups with lower adherence, was associated with a lower risk of developing HF, with a relative risk of 0.79 (95% CI 0.68–0.93, *p* = 0.004) [28].

The male cohort, composed of 37,308 healthy individuals, identified 146 deaths from HF and 1269 cases of HF during a follow-up of 10.9 years. Adherence to the diet was inversely associated with risk of HF development, and the multivariate relative risk of HF mortality was 0.55 (95% CI 0.31–0.98, *p* = 0.007) and for HF, incidence was 0.69 (95% CI 0.57–0.83, *p* < 0.001) [29].

The fourth cohort, with 1000 individuals of both sexes with coronary artery disease, obtained a total of 459 patients who had systolic and diastolic ventricular dysfunction with a reduced ejection fraction (EF < 40%). The score on adherence to the Mediterranean diet was lower in individuals who developed ventricular dysfunction compared to that of the rest of the patients (17.8 ± 3 vs. 19.5 ± 4.7, *p* < 0.001). In addition, better adherence was associated with a lower probability of developing ventricular dysfunction after ischemic event, with an OR of 0.93 (95% 0.88–0.99), cardiac remodeling, with an OR of 0.90 (95% 0.78–1.03), and recurrent events during the two-year follow-up, with an OR of 0.88 (95% 0.80–0.98), even after adjusting for several confounding factors as well as the use of angiotensin-converting enzyme inhibitors [27].

### 3.4. DASH Diet

#### 3.4.1. Definition in the Included Studies

All DASH diet studies used a method introduced by Fung et al. [40] to evaluate dietary adherence. The score is composed of eight components between foods and nutrients: (1) fruits; (2) vegetables; (3) whole grains; (4) nuts and legumes; (5) low fat dairy products; (6) red and processed meats; (7) sweetened beverages; and (8) sodium. For the favorable food groups (fruits, vegetables, whole grains, nuts and legumes, and low-fat dairy products) the highest quintile gets 5 points and the lowest quintile gets 1 point. For unfavorable food groups (red and processed meats, sugary drinks, and sodium), the reverse quintile score is applied. The quintile indices are then summed to obtain a DASH component standard score (theoretical range, 8–40). Higher scores indicate greater adherence to the DASH diet.

#### 3.4.2. DASH Diet and HF

Among the 12 studies analyzed, three cohorts had data on DASH-style diet and its relation to the risk of HF. Del Gobbo et al. did not find a relation of this diet with the incidence of HF [30]; although two other studies found a significant association even after adjusting for other variables [31,32].

The elderly cohort, composed of 61% women, identified 1380 cases of HF in 21 years of follow-up. After adjusting for demographic and lifestyle variables, the DASH pattern was not associated with the incidence of HF, the highest quintile adhering to this diet presenting a relative risk of 1.05 (0.88–1.26, *p* = 0.36) [30].

The cohort with postmenopausal Swedish women obtained 443 cases of HF in seven years of follow-up. Women who had a higher adherence to the DASH diet had a 37% lower rate of HF, i.e., a relative risk of 0.63 (95% CI 0.48–0.81, *p* < 0.001) [31]. The Swedish male cohort identified 807 cases of HF over nine years of follow-up. Subjects classified in the highest quartile for adherence presented a relative risk of 0.78 (95%, 0.65–0.95, *p* = 0.006), that is a 22% lower rate of HF incidence [32].

A cross-sectional study was conducted to evaluate adherence to the DASH diet and its relation to markers of cardiac function in patients from four ethnic groups without cardiovascular disease. Adherence to the DASH diet was significantly associated with diastolic volume and systolic volume when adjusted according to model 1 (sex, age, schooling, body mass index (BMI), smoking, high density lipoprotein (HDL), LDL, diabetes mellitus, systolic blood pressure, use of diabetes medications, antihypertensive, alcohol consumption, and physical activity). The increase of one point in the adherence questionnaire was associated with an increase of 0.31 mL in the final diastolic volume and increases of 0.12 mL/m^2^ in systolic volume. When further adjusted for models 2 (additional adjustment for energy intake) and 3 (adjusted for race/ethnicity), the associations remained significant. Regarding the ejection fraction in model 3, the increase of one point in the adherence questionnaire was associated with an increase of 0.04%, but was marginally significant (*p* = 0.08) [33].

### 3.5. Other Diets and Markers of Cardiac Function

The RCT performed by Anderson et al. [34] investigated the effect of the Paleolithic diet compared to the Nordic recommendations in postmenopausal women. A total of 68 women were followed up with for two years. No significant change was observed in pro BNP natriuretic peptide values at the end of follow-up. A sustained reduction in left ventricular mass was observed in both dietary groups at the six month follow-up. There was no significant change in ejection fraction in both groups. The values shown in Table 1 were estimated from the graphs presented in the article.

An abstract of a cross-sectional study by Pai et al. [35], who evaluated the effects of the vegetarian diet on left ventricular (LV) diastolic function, was found in the search. After adjustments the vegetarian diet, compared to the non-vegetarian diet, was associated with a lower prevalence of LV diastolic dysfunction with an OR of 0.42 (95% 0.24–0.73) and a lower prevalence of LV hypertrophy with OR of 0, 30 (95% 0.08–0.86).

## 4. Discussion

Our results showed that adhering to a Mediterranean or DASH type diet may be advantageous for primary prevention of HF. The results from RCTs showed a protective effect of the Mediterranean diet on the incidence of HF or worsening of cardiac function parameters for patients with previous cardiovascular disease. No studies with low-carb and low-fat diets were found.

Tuttle et al. [22] did not find a significant difference between the Mediterranean diet compared to the low-fat diet on the incidence of HF, but both diets were protective when compared to a control group that followed only usual recommendations. However, Lorgeril et al. [25] demonstrated a decrease in secondary outcomes, including HF development, in patients in the Mediterranean diet group, but the author did not make it clear whether this decrease was significant or not. Fitó et al. [24] did the only RCT that indicates the efficacy of the Mediterranean diet in patients with high cardiovascular risk in improving markers of cardiac function. 

In this sample of adults at high cardiovascular risk, by Papadaki et al. [23], the Mediterranean diet did not result in a lower incidence of HF when compared to the low-fat diet. However, the authors conclude that this pre-specified secondary analysis of the PREDIMED study may have been underpowered, taking into account the small number of observed HF events, to provide valid conclusions.

Cohort studies also showed a clearer result on the efficacy of the Mediterranean diet in both patients with coronary artery disease (CAD) and in previously healthy patients. In addition, the three cohorts showed that the group that adhered strongly to the Mediterranean diet had a lower risk of developing HF when compared to those who adhered more superficially.

Although there are differences between the studies in relation to the standard Mediterranean diet, mainly in relation to the type of addition oil which varied between olive oil and canola oil, the basis of the diet was similar when considering natural foods, whole grains, and fish consumption.

The beneficial effects of olive oil have been attributed to its high content of oleic acid, a type of monounsaturated fatty acid (MUFA), as it protects against oxidative damage [41]. In addition to MUFA, phenolic compounds also exhibit antioxidant and anti-inflammatory effects commonly associated with the origin of major chronic diseases [41,42]. As one of the major phenolic compounds present in virgin olive oils, hydroxytyrosol presents a variety of pharmacological activities, such as antioxidant properties and anticancer, anti-inflammatory, and neuroprotective activities, and beneficial effects on the cardiovascular system [43]. Recent studies have described the vasoprotective effects of polyphenol consumption via olive oil on blood pressure and linked it to the ability to increase endothelial nitric oxide synthesis and endothelium-derived hyperpolarization factor-mediated response [44,45].

It is well known that the inclusion of fish in the diet while excluding red meat, is capable of promoting benefits to human health, especially because fish is lean meat. However, the results of studies that try to point out improvements achieved by the increase in fish consumption and the attempt to correlate this consumption with the increase in omega-3 intake are still inconclusive [46].

For the DASH diet, the results are still conflicting regarding its effectiveness for prevention of HF, even though all included studies evaluated the diet according to the same score. The cohort conducted by Del Gobbo et al. [30] presented unexpected results, demonstrating that such a dietary pattern (DASH diet) was not associated with the incidence of HF. However, after analysis by dietary components, it was observed that higher sodium intake was associated with a significantly increased risk of HF among those participants who had CAD at baseline. A follow-up study of 19 years verified a relationship between diet without sodium restriction and incidence of HF. It was an epidemiological study involving 10,362 normal or overweight individuals with no history of HF. The results pointed to high sodium intake as an independent risk factor for HF and left ventricular hypertrophy in overweight individuals [47].

Left ventricular (LV) function, particularly decreased end-diastolic volume, has been described as an independent risk factor for the future development of HF and cardiac death [48,49,50]. Despite the high relative risk of LV dysfunction, the actual risk of this for HF depends on multiple parameters, especially long-standing hypertension [50,51]. The potentially beneficial association between the DASH diet and LV parameters (and HF) may be the result of some of its food components and low intake of sodium.

The DASH-sodium trial conducted by Sacks et al. [12] strengthens the need to reduce sodium intake to prevent CVD. In this study, the DASH dietary pattern was evaluated combined with three different levels of sodium: low (1500 mg/day), intermediate (2400 mg/day), and high (3300 mg/day). The greatest reductions in blood pressure occurred when the DASH diet was associated with sodium reduction (1500 mg/day). Patients of African descent and those with hypertension achieved the greatest reductions in blood pressure, but the blood pressure reductions also occurred in subjects without hypertension. Thus, there is a significant clinical and public health relevance for the promotion of a DASH-style diet with reduced sodium intake to prevent CVD.

In addition to reducing sodium levels, the DASH dietary pattern involves increased intakes of whole grains, dietary fiber, unsaturated fatty acids, and plant proteins compared to typical Western diets [11]. It also promotes the consumption of foods rich in vitamins (vitamin C and folate), minerals (K, Ca, Mg, and P), amino acids (arginine), and other substances with biological activity in human cells [52]. All these factors may contribute to the beneficial effects of the DASH diet on cardiovascular risk factors. The protective effects of the DASH diet may be due to the combined effects of these molecules on multiple physiological mechanisms, including modification of antioxidant capacity [53], inflammatory response, liver function, coagulation [54], natriuresis [55], sympathetic activation [56], and endothelial function [57].

Regarding markers of cardiac function, two studies evaluated the effects of Mediterranean [24] and Paleolithic [34] diets on BNP and N-terminal (NT) pro BNP levels. Following a Mediterranean-type diet decreased NT-pro BNP natriuretic peptide after one year of intervention. BNP is a sensitive marker and predictor of acute heart failure with a preserved or reduced ejection fraction, and its release is considered to be primarily determined by diastolic dysfunction [58]. Recently, NT-pro BNP has also been shown to be prognostic for mortality and first major cardiovascular events beyond traditional risk factors [59]. Therefore, these results provide further evidence that following a Mediterranean-type diet is useful against the development of CVD.

Other types of diets have also been tested for primary prevention of HF. The vegetarian diet showed a positive effect on the reduction of LV diastolic dysfunction and a lower prevalence of LV hypertrophy. Finally, the RCT performed by Anderson et al. [34] on Paleolithic diet revealed sustained reduction of left ventricular mass, but without significant change in ejection fraction.

Consuming large amounts of vegetables and fruits, which characterizes most vegetarian diets, also brings beneficial effect to the cardiovascular system. Herbal foods provide the body with a variety of antioxidants which inhibit oxidation of LDL cholesterol, raise HDL cholesterol, and reduce total cholesterol concentrations in the circulation [60].

It is known that chronic non-communicable diseases, such as cardiovascular diseases, are complex and multifactorial diseases and not simply developed by deficiency or excess of a certain nutrient. It is believed that regular consumers of whole foods are at a markedly reduced risk of ischemic heart disease, diabetes, and some cancers, sustaining the idea that food synergies play an important role in the prevention of chronic diseases [61].

There is considerable evidence of food synergies and their influence on health. The synergistic effect is not surprising given the complexity of food patterns and food [62].

## 5. Limitations

We acknowledge that data on adherence to dietary patterns and the risk of developing HF are still incomplete. Due to heterogeneity of the studies, it was not possible to make the meta-analysis. Some quality parameters of the included RCTs were flawed, with omission of important information about the generation of sequence and allocation of subjects in the intervention groups. Moreover, regarding the Mediterranean diet studies, the methods used are heterogeneous with respect to the adopted pattern, with different scores being used, which can influence the adherence results. Although the dietary patterns identified may have had some common basic characteristics, each diet has been influenced by different cultural contexts.

## 6. Conclusions

The effect of the investigated dietary patterns in the primary prevention of HF in adult individuals is not totally clear. It is possible that both DASH and Mediterranean diets may contribute to reduced incidence of HF and a decreased morbidity and mortality in adult patients, but these results need to be analyzed with caution due to the quality of evidence found. There is a need for further investigation on different dietary patterns to elucidate their relationship to primary prevention of HF. 

## Figures and Tables

**Table 1:** Characteristics of studies.

Study	SD	Condition or Cohort Name	*N*	Age	Intervention	Control	Duration of Intervention	Outcome
**Mediterranean Diet**								
Tuttle et al., 2008 [22]	RCT	Patients after a first myocardial infarction	201	Mediterranean diet: 58 ± 10 Low-fat diet: 58 ± 9 Usual diet: 57 ± 10	Mediterranean diet or Low-fat diet	Usual diet	2 years	**HF development:** Control: 3 patients Intervention: 0 patients (*p* < 0.25) **Outcome-free survival:** Control: 61 patients Intervention: 85 patients OR: 0.28 (95% 0.13 to 0.63, *p* < 0.002)
Papadaki et al., 2017 [23]	RCT	High risk of cardiovascular disease	7403	TMD + VOO: 67.0 ± 6.2 TMD + nuts: 66.7 ± 6.1 Low-fat: 67.3 ± 6.3	TMD + VOO or TMD + nuts	Low-fat	4.8 years	**HF development:** TMD + VOO: 29 patients TMD + nuts: 33 patients Low-fat: 32 patients TMD + VOO vs. control: HR 0.68 (CI 95% 0.41–1.13) TMD + nuts vs. control: HR 0.92 (CI 95% 0.56−1.49)
Fitó et al., 2014 [24]	RCT	High risk of cardiovascular disease	930	TMD + VOO: 66.4 ± 5.7 TMD + nuts: 66.2 ± 6.0 Low-fat: 67.6 ± 6.1	TMD + VOO or TMD + nuts	Low-fat	1 year	**NT-proBNP (pg/mL)** TMD + VOO vs. control: −70.3 (−133, −7.37) *p* = 0.029 TMD + nuts vs. control: −84.7 (−145, −24.5) *p* = 0.006 **LDL (mg/dL)** TMD + VOO vs. control: −8.27 (−13.9, −2.6) *p* = 0.004 TMD + nuts vs. control: −4.20 (−9.82, −1.42) *p* = 0.145 **Lipoprotein (a) (mg/dL)** TMD + VOO vs. control: −4.17 (−8.12, −0.23) *p* = 0.038 TMD + nuts vs. control: −2.62 (−6.36, −1.13) *p* = 0.170
Lorgeril et al., 1999 [25]	RCT	Patients after a first myocardial infarction	423	NR	Mediterranean diet	Prudent Western-type diet	4 years	**HF development:** Control: 11 patients Intervention: 6 patients **Composite Outcomes(CO1:cardiac deaths and nonfatal AMI):** Control: 44 patients Intervention: 14 patients (*p* = 0.0001) **Composite Outcomes (CO2: CO1 + HF, Stroke, unstable angina, pulmonary embolism or peripheral embolism):** Control: 90 patients Intervention: 27 patients (*p* = 0.0001)
Wirth et al., 2016 [26]	Cohort	European Prospective Investigation into Cancer and Nutrition (EPIC)-Potsdam	24,008	35 to 65	Mediterranean diet	NA	8.2 years	**HF development:** 209 patients Q1 (0–2 points): 1 Q2 (3–4 points): 1.00 (0.70–1.43) Q3 (5–7 points): 0.66 (0.41–1.08)
Chrysohoou et al., 2010 [27]	Cohort	Patients after an acute coronary event	1000	EF < 40%: Male: 64 ± 14 Famale: 71 ± 12 EF ≥ 40%: Male: 62 ± 12 Famale: 67 ± 12	Mediterranean diet	NA	2 years	**Left ventricular systolic** **dysfunction:** OR 0.93 (0.88–0.99) **Ventricular remodeling:** OR 0.90 (0.78–1.03) **Re-current events ACS:** OR 0.88 (0.80–0.98)
Tektonidis et al., 2015 [28]	Cohort	Swedish mammography cohort	32,921	48 to 83	Mediterranean diet	NA	10 years	**HF development:** Total: 1648 patients Q4: 244 patients RR 0.79 (0.68–0.93, *p* = 0.001)
Tektonidis et al., 2016 [29]	Cohort	Cohort of Swedish men	37,308	45 to79	Mediterranean diet	NA	11 years	**HF development:** Total: 1269 patients Q4: 169 patients RR 0.69 (0.57–0.83, *p* < 0.001) **HF mortality** Total: 146 Q4: 16 patients RR 0.55 (0.31–0.98, *p* = 0.007)
**DASH Diet**								
Del Gobbo et al., 2015 [30]	Cohort	Cardiovascular health study of elderly patients with and without CVD	4490	Q1: 72.2 ± 5.3 Q5: 72.0 ± 5.1	DASH	NA	21 years	**HF development:** Total: 1380 patients Q5: 235 patients RR 1.05 (0.88–1.26, *p* = 0.36)
Levitan et al., 2009a [31]	Cohort	Swedish mammography cohort	36,019	48–83	DASH	NA	7 years	**HF development:** Total: 443 patients Q4: 89 patients RR 0.63 (0.48–0.81, *p* < 0.001)
Levitan et al., 2009b [32]	Cohort	Cohort of Swedish men	38,987	45 to 79	DASH	NA	9 years	**HF development:** Total: 807 patients Q4: 192 patients RR 0.78 (0.65–0.95, *p* = 0.006)
Nguyen et al., 2012 [33]	Cross-sectional	Multi-ethnic group free of clinical cardiovascular disease (CVD)	4506	45 to 84	DASH	NA	NA	**A 1-unit increase in the DASH:** Model 1 ^1^: End-diastolic volume: 0.31 (0.08), *p* < 0.001 Stroke volume: 0.12 (0.03), *p* < 0.001 LVEF: 0.03 (0.02), *p* = 0.15 Model 2 ^2^ End-diastolic volume: 0.31 (0.08), *p* < 0.001 Stroke volume: 0.12 (0.03), *p* < 0.001 LVEF: 0.03 (0.02), *p* = 0.15 Model 3 ^3^ End-diastolic volume: 0.26 (0.08), *p* < 0.01 Stroke volume: 0.10 (0.03), *p* < 0.001 LVEF: 0.04 (0.02), *p* = 0.08
**Paleolithic Diet**								
Andersson et al., 2016 [34]	RTC	Healthy postmenopausal women	68	NR	Paleolithic diet (30% PTN, 40% LIP, 30% CHO)	Nordic nutrition recommendation (15% PTN, 25–30% LIP, 55–60% CHO)	2 years	**NT-proBNP (pg/mL)** Group Paleolithic diet: Baseline: 64.4 (6.1) After 24 months: 87.6 (18.6) Group Nordic Nutrition Recommendation: Baseline: 47.3 (4.8) After 24 months: 67.9 (9.6) (*p* = 0.764) **Left ventricular mass** (g) Group Paleolithic diet: Basline: 101 After 24 months: 90 Group Nordic Nutrition Recommendation: Baseline: 107 After 24 months: 99 (*p* < 0.05)
**Vegetarian Diet**								
Pai et al., 2015 [35]	Cross-sectional	Adventist health study-2	206	74.0 ± 10.0	Vegetarian diet	non-vegetarian diet	NA	LV diastolic dysfunction: OR 0.42 (IC 95% 0.24–0.73) LV hypertrophy: OR 0.30 (IC 95% 0.08–0.86)

ACS: Acute coronary syndrome; AMI: acute myocardial infarction; BNP: Brain-Type Natriuretic Peptide; CHO: carbohydrate; CVD: cardiovascular disease; DASH: dietary approaches to stop hypertension; DM: diabetes mellitus; EF: Ejection fraction; HF: Heart failure; LIP: lipids; HR: hazard ratio; LV: left ventricle; LVEF: ejection fraction of the left ventricle; NA: not applicable; NR: not reported; NT: N-terminal; PTN: protein; RCT: randomized clinical trial; RR: relative risk; SD: study design; TMD+VOO: Traditional Mediterranean diet + olive oil. ^1^ (sex, age, schooling, body mass index (BMI), smoking, HDL: high density lipoprotein, LDL: low density lipoprotein, diabetes mellitus, systolic blood pressure, use of diabetes medications, use of blood pressure lowering medications, alcohol intake and physical activity); OR: odds ratio; RR: relative risk. ^2^ (model 1 + energy consumption). ^3^ (model 2 + race/ethnicity).

**Table 2:** Quality assessment of studies.

Study	SD	RSG	AC	Blinding of Research	Blinding of PP	Blinding of OA	LE	Do They Describe Confounders in an Adjusted Analysis?	Do You Assess the Balance Between the Groups at the Start of the Study?
Andersson et al.	RCT	High risk	Unclear risk	Unclear risk	Unclear risk	Low risk	Low risk	NA	NA
Fitó et al.	RCT	Low risk	Low risk	Unclear risk	Unclear risk	Low risk	Low risk	NA	NA
Lorgeril et al.	RCT	Unclear risk	Unclear risk	Unclear risk	Unclear risk	Low risk	Low risk	NA	NA
Papadaki et al.	RCT	Low risk	Low risk	Unclear risk	Unclear risk	Low risk	Low risk	NA	NA
Tuttle et al.	RCT	Low risk	Low risk	Unclear risk	Unclear risk	Low risk	Low risk	NA	NA
Chrysohoou et al.	RCT	Low risk	Low risk	Unclear risk	Unclear risk	Low risk	Low risk	NA	NA
Del Gobbo et al.	Cohort	NA	NA	NA	NA	NA	NA	Low risk	High risk
Levitan et al.	Cohort	NA	NA	NA	NA	NA	NA	Low risk	Low risk
Levitan et al.	Cohort	NA	NA	NA	NA	NA	NA	Low risk	Low risk
Tekitonidis et al.	Cohort	NA	NA	NA	NA	NA	NA	Low risk	Low risk
Tekitonidis et al.	Cohort	NA	NA	NA	NA	NA	NA	Low risk	Low risk
Wirth et al.	Cohort	NA	NA	NA	NA	NA	NA	Low risk	Low risk
Nguyen et al.	Cross-sectional study	NA	NA	NA	NA	NA	NA	Low risk	Low risk
Pai et al. *	Cross-sectional study	NA	NA	NA	NA	NA	NA	Low risk	High risk

AC: allocation concealment; LE: losses and exclusions; NA: not applicable; OA: outcome assessment; PP: participants and personnel; RCT: randomized clinical trial; RSG: random sequence generation; SD: study design; (*) abstract.
